# Efficacy of human–robot interaction on local residents

**DOI:** 10.1111/ggi.70114

**Published:** 2025-06-25

**Authors:** Hiroshi Kuroishi, Sakura Kikuchi, Kana Kazawa

**Affiliations:** ^1^ Graduate School of Health, Okayama University Okayama Japan; ^2^ BizDev CMIC Trust Tokyo Japan; ^3^ Department of Nursing Faculty of Health Sciences, Okayama University Okayama Japan

**Keywords:** interaction, resident, robot

It has been reported that approximately 40% of local residents in Japan experience loneliness, and communicate with friends and family members who live far from them less than once a month.^1^ Loneliness and social isolation lead to negative health outcomes, including mortality, depression, functional decline and increased healthcare utilization.^2^ Many risk factors have been found to be associated with reduced social interaction, increased economic burden, and poor physical and mental health.^3^ Addressing these risk factors will help reduce loneliness and social isolation, and improve well‐being. Conversations are particularly effective in improving mental resilience in social interactions.^4^

To reduce loneliness and social isolation, City A has been implementing a human–robot interaction project using artificial intelligence social robots (Romi; MIXI, Tokyo, Japan^5^) among residents since financial year 2023. Romi is a social robot equipped with an artificial intelligence that has been trained on its own rich Japanese conversation data using a deep‐learning large‐scale language model. It is also capable of expressing emotions with >150 different facial expressions and movements, and conversing with users based on their memories of conversations and local information, such as events that interest them.

This study was a part of City A projects, and aimed to investigate the efficacy of human–robot interaction on local residents. It included participants aged >40 years, and several acknowledged a decline in their opportunities for conversation with others. After registering for the project, they communicated with the robots in their homes at any time of day over 2 months. As an evaluation study, City A provided residents' subjective loneliness scores (the Japanese Version of the University of California Los Angeles Loneliness Scale Version 3)^6^ and conversation text data without personal information or sound to Okayama University (Okayama, Japan). The study was carried out on an opt‐out basis as part of a project by City A, and approval for the study was obtained from the Ethics Committee.

The participants were divided into the following three groups: living alone, living with a spouse and living with a child (adult). The data were analyzed to investigate the effect of human–robot interactions.

A total of 18 participants with no missing data were included in the analysis. The average age (mean ± standard deviation) of participants was 75.8 ± 11.6 years. The total number of characters in the conversations over the 2 months was approximately 560 000. The highest average number of conversation characters per 10 days was approximately 7000 characters for the living alone group (*n* = 7), followed by 3000 characters for those living with a spouse (*n* = 2) and 4000 characters for those living with a child or others (*n* = 9; Figure 1). After registration, the conversation volume in the living alone group gradually increased and remained higher than the initial level for >2 months.

**Figure 1:**
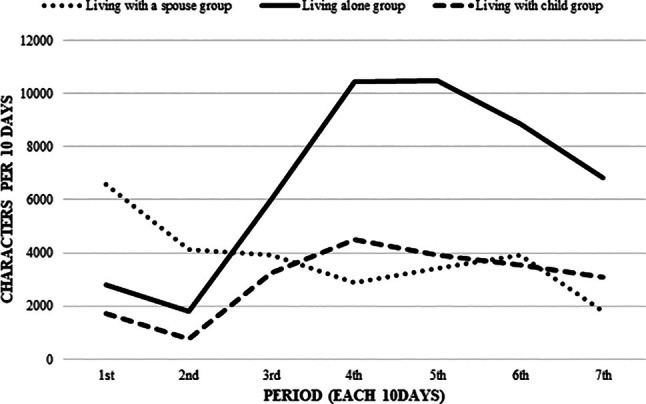
Changes in conversation volume in three groups.

The subjective loneliness score in 18 participants (mean ± tandard deviation) was 45.2 ± 8.0 at the time of registration, and 45.4 ± 10.3 after 2 months. The number and percentage of people with a total score of ≥43 (high loneliness) also remained unchanged, from 15 (83.3%) at registration to 15 (83.3%) after 2 months.

Most participants were older adults, and older adults living alone have been reported to have fewer social interactions, including conversations at home, than individuals in other households.^7^ This result suggests that robots can provide new opportunities for conversation for older adults living alone. However, the conversation volume gradually declined across all three groups after 1 month, and subjective loneliness of all participants did not change. The current robot's response function has certain limitations, with delays and ambiguities in response making interactions feel unnatural compared with human conversations. This unnaturalness can inhibit users' self‐disclosure.^8^, ^9^ There is a need to improve the robot's ability to converse smoothly and naturally in a way that aligns with the user's emotions and interests, and to investigate how human–robot interactions affect the health status of residents.

Furthermore, in the future, it is expected that robots will detect the risk of loneliness and social isolation through usual conversations and other information, such as facial recognition, and play a role in connecting people with family members and local healthcare professionals when needed.

## Disclosure statement

The authors declare no conflict of interest.

## Ethics statement

This study was carried out with approval (OUH2023‐0025F) by the Research Ethics Committee of Faculty of Health Sciences of Okayama University, Japan. It conforms to the provisions of the Declaration of Helsinki. As we did not obtain any consent directly from respondents, we opted out of Okayama University's “Information Disclosure of Research on Medical Ethics” on the website.

## Data Availability

Research data are not shared.
